# Patient Perspectives on AI- and XR-Enabled Telemedicine: A Cross-Sectional Survey in Romania

**DOI:** 10.3390/healthcare13212672

**Published:** 2025-10-23

**Authors:** Codrina Mihaela Levai, Laura Alexandra Nussbaum, Daian-Ionel Popa, Sonia-Roxana Burtic, Bogdan Florin Căpăstraru, Iulius Jugănaru

**Affiliations:** 1 Doctoral School, Faculty of Medicine, “Victor Babes” University of Medicine and Pharmacy, 300041 Timisoara, Romania; codrinalevai@umft.ro (C.M.L.); daian-ionel.popa@umft.ro (D.-I.P.); dr.soniaburtic@umft.ro (S.-R.B.); bogdan.capastraru@umft.ro (B.F.C.); 2 Research Center for Medical Communication, “Victor Babes” University of Medicine and Pharmacy of Timisoara, 300041 Timisoara, Romania; 3 Department of Neurosciences, “Victor Babes” University of Medicine and Pharmacy of Timisoara, 300041 Timisoara, Romania; 4 Department XI Pediatrics, Discipline I-Pediatrics, “Victor Babes” University of Medicine and Pharmacy of Timisoara, 300041 Timisoara, Romania; juganaru.iulius@umft.ro; 5 Department of Research Center for Disturbances of Growth and Development in Children–BELIVE, “Victor Babes” University of Medicine and Pharmacy of Timisoara, 300041 Timisoara, Romania

**Keywords:** telemedicine, artificial intelligence, virtual reality, patient acceptance of health care, data privacy

## Abstract

**Background and Objectives**: As artificial intelligence (AI) and extended reality (XR) enter routine care, understanding patient acceptance is essential. We assessed attitudes toward AI/XR-enabled telemedicine among Romanian patients and examined correlates of acceptance. **Methods**: We analyzed 198 survey responses to a 20-item questionnaire. Ordinal items were encoded 1–4. The Acceptance Index measured trust in AI, perceived improvement in care, and willingness to choose AI-assisted visits (on a 1–4 scale). **Results**: Respondents were predominantly 31–50 years old (62.6%) and university educated (76.2%); 27.3% reported prior experience with AI/XR. Acceptance averaged 3.27 ± 0.59 (α = 0.780) and did not differ by age (*p* = 0.922). Prior users showed higher acceptance than non-users (3.47 ± 0.47 vs. 3.19 ± 0.59; *p* = 0.0011). Knowledge (ρ = 0.189, *p* = 0.048) and perceived accessibility (ρ = 0.229, *p* = 0.016) correlated with acceptance; privacy concern did not (ρ = 0.072, *p* = 0.455). Subgroups: Prior use was associated with higher acceptance across education levels, with a significant effect in secondary education (Holm-adjusted *p* = 0.029; Cliff’s δ = 0.56). Ordinal logistic model: higher willingness to pay (OR 6.81, 95% CI 3.39–13.66, *p* < 0.001) and greater perceived accessibility (OR 1.83, 95% CI 1.03–3.24, *p* = 0.040) independently predicted choosing AI-assisted visits. **Conclusions**: Patient acceptance of AI/XR-enabled telemedicine was moderate to high, strongest among prior users, and increased when access felt easy. Knowledge modestly supported acceptance; privacy concerns did not diminish it. Clear value propositions, streamlined access, and optional exposure pathways may enhance informed uptake.

## 1. Introduction

Telemedicine has moved from a crisis stopgap to a durable layer of care delivery, with sustained post-pandemic use across outpatient specialties and measurable patient-reported benefits, including convenience and satisfaction [1,2,3,4]. Evidence syntheses suggest teleconsultations can substitute for, or complement, in-person visits without compromising many process and outcome metrics, particularly when protocols and patient selection are explicit [3,4]. These shifts form the substrate on which newer capabilities, artificial intelligence (AI) and extended reality (XR), are now being integrated into remote care.

Yet uptake remains uneven. Utilization patterns vary by geography and service line, with U.S. health-center data showing rapid scaling but heterogeneous intensity across regions and time [5]. A persistent “digital readiness” gap among older adults, driven by device access, sensory/functional limitations, and limited technology experience, continues to shape who benefits from telemedicine [6]. Rural communities also face bandwidth constraints and service fragmentation, influencing both access and outcomes from remote interventions [7]. These equity and infrastructure realities are central when asking patients to evaluate additional layers of digital care, such as AI or XR. Examples in telemedicine include AI-supported symptom triage and routing in virtual front-doors and AI-assisted image interpretation in teledermatology. XR applications include home-based virtual-reality telerehabilitation sessions and augmented-reality telementoring, where a remote clinician overlays guidance during live video consultations.

In parallel, AI has matured from proof-of-concept to deployment in imaging triage, symptom assessment, and decision support; the promise is “high-performance medicine” that augments clinicians while streamlining workflows [8]. Still, patients weigh performance against comprehensibility. Choice-based experiments show that people often trade off model accuracy for a desire to understand recommendations [9]. At the same time, leading perspectives caution that popular explainability techniques may not reliably confer safety or trust, urging rigorous validation and transparent evaluation over post hoc rationales [10,11]. Together, these strands suggest that patient acceptance will depend on demonstrable benefits, appropriate oversight, and communication that aligns with health literacy.

XR—which includes virtual and augmented reality, adds sensorimotor fidelity to remote encounters. Beyond provider telementoring and procedural guidance with augmented reality overlays [12], randomized and systematic evidence indicates that virtual reality (VR)-supported rehabilitation can modestly improve functional outcomes in neurological recovery compared with conventional therapy, while also enhancing engagement, an important consideration for long-term condition management [13]. Early applications in patient education and analgesia further suggest XR can influence adherence and perceived value (perceived clinical and experiential benefit relative to monetary cost, time, and effort), positioning it as a candidate amplifier of telemedicine’s effectiveness [14].

These opportunities carry design and safety considerations. Cybersickness (nausea, oculomotor strain, disorientation) is not rare and varies by headset characteristics, content motion, and exposure time; mitigation requires deliberate scenario design, session titration, and accessibility accommodations [15]. Patient-centered XR in telemedicine must, therefore, prioritize tolerability and inclusivity alongside efficacy. From a cost perspective, XR devices and content licensing are variably reimbursed; coverage frequently depends on program-level funding or pilot status, with patient out-of-pocket exposure influenced by local payer policies and device procurement models.

Barriers to digital care adoption, including privacy concerns, workflow fit, training needs, and reimbursement uncertainty, are well-documented and remain germane to AI/XR-enabled services [16]. In Europe, the evolving regulatory environment adds context: the EU Artificial Intelligence Act introduces risk-tiered obligations for medical AI systems, emphasizing transparency, data governance, and post-market monitoring; concurrently, the GDPR frames consent, purpose limitation, and data-subject rights in ways patients notice and respond to when judging trustworthiness [17,18]. Clear communication about the model’s scope and limits, as well as data handling, is therefore integral to acceptance.

Romania offers a salient setting. Telemedicine accelerated during the COVID-19 pandemic and continues to evolve within national services, but its uptake reflects heterogeneity in infrastructure and digital literacy across regions and age groups [19]. Importantly, recent Romanian work using the Technology Acceptance Model among physicians highlights perceived usefulness, social norms, and access to records as key drivers of behavioral intention to use telemedicine [20].

While telemedicine acceptance is well studied internationally, little is known about AI/XR-specific perceptions among Romanian patients. We hypothesized that prior exposure and perceived accessibility would be positively associated with acceptance and that privacy concern would not materially reduce acceptance. Our objective was to quantify acceptance of AI/XR-enabled telemedicine and examine these correlates.

## 2. Materials and Methods

### 2.1. Study Design, Setting, and Ethics

This cross-sectional study was conducted in a university-affiliated health-system setting in Western Romania. Ethical approval was granted by the “Institutional Review Board of Victor Babes University of Medicine and Pharmacy, Timisoara, affiliated with the “Pius Brinzeu” Clinical Emergency Hospital, Timisoara, Romania. The Local Commission of Ethics for Scientific Research of the Victor Babes University of Medicine and Pharmacy from Timisoara, Romania, operates under article 167 provisions of Law no. 95/2006, art. 28, chapter VIII of order 904/2006; with EU GCP Directives 2005/28/EC, International Conference of Harmonisation of Technical Requirements for Registration of Pharmaceuticals for Human Use (ICH); and with the Declaration of Helsinki—Recommendations Guiding Medical Doctors in Biomedical Research Involving Human Subjects. Approval number 2429 from 10 January 2023.

We conducted a cross-sectional, anonymous online survey targeting adult patients (aged 18 years or older) residing in Romania. The questionnaire consisted of 20 items and was administered in Romanian via a web form; data were collected February–July 2024; data were collected over a six-month period. Participation was voluntary, without incentives, and respondents provided informed consent by proceeding past the survey introduction. No directly identifying information was analyzed; any contact fields present in the form export were removed prior to analysis. The study was undertaken for research training and methods development; the analytic plan, variable codings, and exclusion rules were specified prior to running inferential tests. Reporting follows Strengthening the Reporting of Observational Studies in Epidemiology (STROBE) guidance for cross-sectional surveys [21].

Recruitment used a non-probability, convenience approach. Invitations with a survey link/QR code were posted on university-affiliated outpatient clinics’ notice boards and patient portal pages, shared on institutional social-media channels, and circulated via community groups. The target population was adult residents of Romania; respondents were not restricted to any diagnosis and thus represent a general (not disease-specific) patient population engaging with a university-affiliated health system’s public channels. The form required completion of mandatory items prior to submission; partial attempts were not stored, and platform analytics did not log view-only sessions, precluding calculation of a denominator-based completion rate.

### 2.2. Eligibility and Instrument Development

Eligibility criteria were age ≥ 18 years and completion of all required items for the relevant analyses. Items assessed demographics, prior use of AI/XR-assisted health services, self-rated knowledge about AI/XR, trust in AI-assisted diagnosis/treatment, perceived improvement of care with AI/XR, privacy concern, perceived accessibility of AI/XR-enabled telemedicine, willingness to choose an AI-assisted visit, willingness to pay, cost–benefit appraisal, and information needs. The instrument framed ‘AI/XR-enabled telemedicine’ as a combined concept for evaluation; we did not randomize or separate AI-specific versus XR-specific vignettes, so modality-specific effects could not be isolated.

Ordinal items were harmonized to 4 response levels where conceptually appropriate and encoded such that higher values reflect more favorable perceptions (1 = least favorable to 4 = most favorable), except for Privacy Concern, for which higher scores reflect greater concern (less favorable). The Acceptance Index was computed as the mean of three items—Trust in AI, Perceived Improvement, and Willingness to Choose an AI-assisted visit—yielding a composite score of 1–4. Internal consistency of the composite was evaluated via Cronbach’s alpha.

Survey framing and examples: To ground responses in telemedicine use cases, the introduction provided short examples (AI triage and follow-up routing; AI-assisted image interpretation during a video visit; VR-assisted home telerehabilitation; and AR guidance during a remote consult). Respondents were informed that a clinician remained responsible for care decisions. No hands-on intervention occurred.

Conceptual framework: The Acceptance Index (trust, perceived improvement, and willingness to choose) maps to perceived usefulness and attitude (Technology Acceptance Model) and to performance/effort expectancy and behavioral intention (UTAUT). Perceived accessibility approximates facilitating conditions, and willingness to pay reflects perceived value. These anchors informed variable selection a priori.

Instrument provenance and validity: The questionnaire was newly developed from prior literature and expert input (telemedicine, AI, and XR clinicians; n = 4) for face/content validity. Cognitive pretesting with volunteers (n = 8) informed wording refinements. Internal consistency for the Acceptance Index was acceptable (α = 0.780).

### 2.3. Outcomes, Predictors, and Subgroup Definitions

Primary outcome: Acceptance Index (1–4). Secondary outcomes: each component item (Trust, Perceived Improvement, Choose AI Visit), Perceived Accessibility (1–4), Privacy Concern (1–4; higher = worse), Willingness to Pay (1–4), and Cost–Benefit appraisal. Key predictors/covariates: self-rated Knowledge (1–4), Prior AI/XR Use (Yes/No; “Don’t know” excluded for two-group tests), and education (primary, secondary, undergraduate, postgraduate). Age was analyzed in a priori bands (21–30, 31–40, 41–50, 51–60, 61–70, 71–80). Subgroups: prespecified comparisons by age band and prior use; exploratory analyses stratified by education level.

### 2.4. Statistical Analysis

Categorical variables are summarized as counts and percentages; continuous/ordinal composites are summarized as mean ± SD and, where helpful, median [IQR]. Group differences across ≥ 3 ordered groups used Kruskal–Wallis tests; two-group comparisons used Mann–Whitney U. Rank-based associations between ordinal variables used Spearman’s ρ. For 2 × 2 tables, we used Pearson’s χ^2^ with continuity correction where sparse. Alongside *p*-values, we report effect sizes appropriate to the test: ε^2^ for Kruskal–Wallis, Cliff’s δ for Mann–Whitney U, and Cramér’s V for χ^2^. Two-sided α = 0.05 defined statistical significance; 95% confidence intervals accompanied effect sizes where feasible.

To estimate independent associations with acceptance, we fit an ordinary least squares (OLS) model with the Acceptance Index as the dependent variable. Predictors were Knowledge, Privacy Concern, Perceived Accessibility, Willingness to Pay, Prior Use (dummies for Yes/No and Don’t know), age bands (dummy-coded; 21–30 as reference), and education (dummy-coded; primary as reference). Model diagnostics included inspection of residual–fitted plots, leverage and influence (Cook’s D), and multicollinearity (variance inflation factors); no observations were excluded a priori. We report unstandardized coefficients (β), robust SE, *p*-values, and model R^2^.

To investigate whether the association between prior AI/XR use and acceptance holds across educational levels, we conducted within-stratum Mann–Whitney U tests (Yes vs. No) and reported Cliff’s δ. To control for family-wise error across strata, *p*-values were adjusted using the Holm–Bonferroni method. Because ‘Choose an AI-assisted visit’ is ordinal (1–4), the primary inferential model was a proportional-odds ordinal logistic regression. In secondary analyses, we modeled the continuous Acceptance Index using OLS with robust SEs. Analyses using n = 110 reflect complete-case inclusion for models requiring non-missing covariates; missingness arose from optional items and listwise deletion was applied. We did not impute data.

## 3. Results

The sample (N = 198) skewed toward mid-life adults: 31–40 years (63/198; 31.8%) and 41–50 years (61/198; 30.8%) together comprised 62.6%, followed by 21–30 years (39/198; 19.7%), 51–60 years (24/198; 12.1%), 71–80 years (9/198; 4.5%), and 61–70 years (2/198; 1.0%). Educational attainment was predominantly tertiary, with 104/198 (52.5%) holding an undergraduate degree and 47/198 (23.7%) postgraduate training; 42/198 (21.2%) reported secondary education and 5/198 (2.5%) primary education. Prior experience with AI/XR-enabled health services was reported by 54/198 (27.3%), while 118/198 (59.6%) had not used such services and 26/198 (13.1%) were unsure. Self-rated knowledge clustered in the middle of the scale: “slightly informed” 76/198 (38.4%) and “moderately informed” 72/198 (36.4%); 29/198 (14.6%) were “not informed” and 21/198 (10.6%) “very informed”, as presented in Table 1.

Acceptance metrics were broadly stable across age bands. Mean Acceptance Index (1–4) ranged narrowly from 3.17 ± 0.71 (61–70 years; n = 2) to 3.48 ± 0.34 (71–80 years; n = 9), with intermediate values for 21–30 (3.28 ± 0.60), 31–40 (3.23 ± 0.62), 41–50 (3.28 ± 0.62), and 51–60 (3.26 ± 0.54). Component items showed similar clustering: Trust in AI spanned 3.00 ± 0.73 (21–30) to 3.33 ± 0.50 (71–80), Perceived Improvement ranged from 3.38 to 3.67, and choosing an AI-assisted visit ranged from 3.00 to 3.44. Kruskal–Wallis tests detected no age-group differences (Trust *p* = 0.4565; Perceived Improvement *p* = 0.8746; Choose AI Visit *p* = 0.7111; Acceptance Index *p* = 0.9216). Although the oldest group displayed the highest mean acceptance and perceived improvement (3.48 and 3.67, respectively), these differences were not statistically significant and should be interpreted cautiously given small cell sizes at the tails of the age distribution (e.g., 61–70 years, n = 2; 71–80 years, n = 9), as seen in Table 2.

Prior exposure to AI/XR-enabled services correlated with higher acceptance. Patients reporting prior use (n = 54) had a mean Acceptance Index of 3.47 ± 0.47, compared to 3.19 ± 0.59 among non-users (n = 118), representing a difference of +0.28 points on a 1–4 scale. The Mann–Whitney U test confirmed statistical significance (U = 4157.5; *p* = 0.0011, two-sided), supporting the practical inference that familiarity may be associated with greater acceptance. Given the similar standard deviations (0.47 vs. 0.59), the observed mean gap likely reflects a non-trivial shift in central tendency rather than dispersion, aligning with diffusion-of-innovation patterns in which direct experience reduces uncertainty and increases willingness to adopt (Table 3).

Spearman correlations highlighted modest but coherent associations. Knowledge was positively correlated with perceived accessibility (ρ = 0.322; *p* = 0.0006) and, to a lesser extent, with acceptance (ρ = 0.189; *p* = 0.0478). Privacy concerns (higher = worse) were correlated with knowledge (ρ = 0.259; *p* = 0.0063), but not with accessibility (ρ = 0.141; *p* = 0.1421) or acceptance (ρ = 0.072; *p* = 0.4547), suggesting that privacy worries did not meaningfully dampen acceptance in this cohort. Perceived accessibility showed a small-to-moderate association with acceptance (ρ = 0.229; *p* = 0.0162), as shown in Table 4.

**Figure 1:**
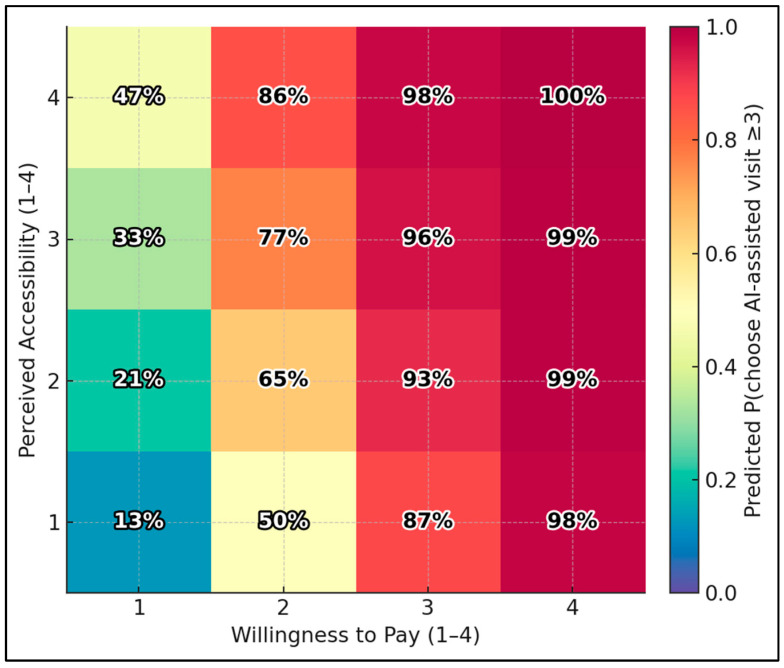
Predicted choice heatmap: accessibility × willingness to pay.

Using the ordinal-logit model, the predicted probability of choosing an AI-assisted visit (score ≥ 3) ranges from 12.8% at the lowest corner (Accessibility = 1, Willingness-to-Pay = 1) to 99.6% at the highest corner (Accessibility = 4, WTP = 4). Holding other variables at medians, boosting accessibility from 1 → 4 with WTP fixed at 1 raises the probability from 12.8% → 47.2%; boosting WTP from 1 → 4 with accessibility fixed at 1 raises it from 12.8% → 97.9% (Figure 1).

Dichotomizing choice into No/Low (1–2) versus Yes/Maybe (3–4) revealed no association with high privacy concern. Among respondents without high concern, 71/85 (83.5%) indicated Yes/Maybe versus 14/85 (16.5%) No/Low. Among those with high concern, 22/25 (88.0%) indicated Yes/Maybe versus 3/25 (12.0%) No/Low. The distributions were nearly identical, and Pearson’s χ^2^ with continuity correction was non-significant (χ^2^ = 0.052; df = 1; *p* = 0.819; total n = 110 for this analysis), as presented in Table 5.

In the OLS model predicting the continuous Acceptance Index (n = 110; HC3 SE; R^2^ = 0.461), willingness to pay more emerged as the only robust predictor (β = +0.432; SE = 0.068; *p* < 0.001), implying a ~0.43-point increase in acceptance per one-level increase on the 1–4 payment scale. Knowledge (β = −0.048; *p* = 0.513), privacy concern (β = −0.131; *p* = 0.325), perceived accessibility (β = +0.078; *p* = 0.308), prior use (No vs. Yes β = −0.104; *p* = 0.401; Don’t know vs. Yes β = −0.005; *p* = 0.983), age bands, and education levels were not significant after mutual adjustment. Intercept was 2.427 (*p* < 0.001). The explained variance (46.1%) indicates moderate model fit, with the economic/value construct (willingness to pay) accounting for a substantial share of the independent association with acceptance (Table 6).

Subgroup analyses by individual level of education revealed a consistent direction toward higher acceptance among prior users, achieving statistical significance only within the secondary education stratum after Holm correction. In secondary education, prior users (n = 10) had a mean acceptance of 3.43 ± 0.39, versus 2.78 ± 0.75 for non-users (n = 24), with a Mann–Whitney U of 187.5, a raw *p*-value of 0.0097, and a Holm-adjusted *p*-value of 0.0291, indicating a moderate effect (Cliff’s δ = 0.563). The undergraduate stratum showed a similar but non-significant pattern (Yes 3.43 ± 0.55 [n = 30] vs. No 3.26 ± 0.54 [n = 63]; *p* = 0.0697; Holm *p* = 0.1394; δ = 0.229). Postgraduates also trended higher (Yes 3.57 ± 0.33 [n = 14] vs. No 3.38 ± 0.41 [n = 27]; *p* = 0.174; δ = 0.254). No primary-education respondents reported prior use (Yes n = 0; No n = 4), as described in Table 7.

In the proportional-odds model for the ordinal outcome “choose an AI-assisted visit” (1–4 scale), willingness to pay more was the dominant independent predictor (OR = 6.807; 95% CI 3.39–13.66; *p* < 0.001), indicating each one-level increase multiplied the odds of choosing higher categories by ~6.8. Perceived accessibility was also independently associated (OR = 1.826; 95% CI 1.03–3.24; *p* = 0.040). Knowledge (OR = 1.214; *p* = 0.514), privacy concern (OR = 0.492; *p* = 0.228), prior use (OR = 0.63; *p* = 0.412), age (OR = 1.085; *p* = 0.659), and education (OR = 0.815; *p* = 0.512) were not significant (Table 8).

**Figure 2:**
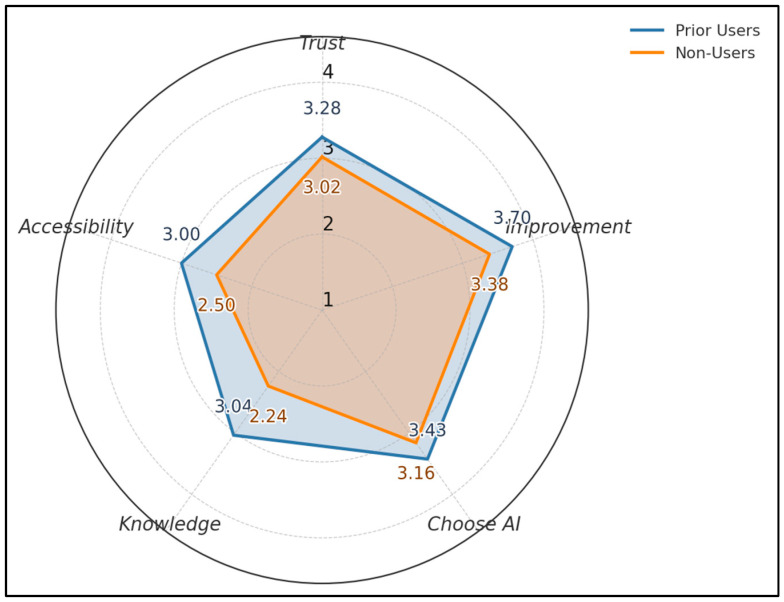
Acceptance profile by prior use.

Across five 1–4 metrics, prior users score higher than non-users: Trust 3.28 vs. 3.02, Perceived Improvement 3.70 vs. 3.38, Choose AI 3.43 vs. 3.16, Knowledge 3.04 vs. 2.24, Accessibility 3.00 vs. 2.50. The largest gap appears in Knowledge (+0.80) and Perceived Improvement (+0.32), as seen in Figure 2.

## 4. Discussion

### 4.1. Analysis of Findings

Our survey shows that Romanian patients report moderate-to-high acceptance of AI/XR-enabled telemedicine (Acceptance Index 3.27 ± 0.59; α = 0.780), with no measurable differences across age groups (all Kruskal–Wallis *p* > 0.45). Notably, 27.3% reported prior AI/XR use and 36.4% self-rated as moderately informed—figures that likely exceed general-population baselines. This may reflect the university-affiliated recruitment channels and higher educational attainment in our sample, and should temper generalizability. This pattern contrasts with concerns that older adults’ “digital readiness” gap necessarily depresses acceptance [6], although the oldest strata were small (61–70 y: n = 2; 71–80 y: n = 9) and estimates are imprecise.

Prior exposure was associated with meaningfully higher acceptance (3.47 ± 0.47 vs. 3.19 ± 0.59; *p* = 0.0011), consistent with diffusion processes in which direct experience reduces uncertainty. Overall levels align with the literature, showing sustained telehealth satisfaction post-pandemic [1,2,3,4,5]. The absence of age effects within this relatively well-educated sample (76.2% tertiary education) suggests that structural barriers, rather than age per se, may be the dominant constraint when they occur. Descriptively, the youngest (21–30 years: mean 3.38) and oldest (71–80 years: mean 3.44) strata showed relatively higher willingness to choose an AI-assisted visit, but differences were not statistically significant and small cell sizes warrant caution.

In the proportional-odds model, willingness to pay was strongly associated with choosing an AI-assisted visit (OR 6.81, 95% CI 3.39–13.66; *p* < 0.001), and perceived accessibility had an independent, albeit smaller, effect (OR 1.83, 95% CI 1.03–3.24; *p* = 0.040). OLS results converged, with willingness to pay being the only significant predictor of the continuous Acceptance Index (β = +0.432; *p* < 0.001; R^2^ = 0.461). Knowledge was correlated modestly with acceptance (ρ = 0.189; *p* = 0.048), but was not independently predictive; privacy concern neither correlated with acceptance (ρ = 0.072; *p* = 0.455) nor shifted choice (χ^2^ = 0.052; *p* = 0.819). Taken together, these findings suggest that clear value propositions and low-friction access can outweigh generalized privacy anxieties—echoing evidence that patients trade off model performance, interpretability, and convenience [9,10,11]. For XR, evidence of benefit in rehabilitation and engagement [13,14] alongside cybersickness risks [15] underscores the need for deliberate content design and session titration to sustain acceptance.

Within the EU, AI deployments are shaped by the AI Act’s risk-tiered obligations and by General Data Protection Regulation (GDPR)-anchored transparency and data-subject rights [17,18]. In Romania, the formalization of telemedicine continues amid uneven infrastructure and digital literacy [19], and physician adoption is driven by perceived usefulness and social norms [21]. Recent efforts to quantify telemedicine acceptance among Romanian patients with diabetes include the development and validation of a structured instrument by Patrascu et al., which demonstrated strong reliability and utility for assessing patients’ desirability for, acceptability of, and adherence to telemedicine. Nevertheless, social desirability effects (the tendency to provide favorable responses because AI/XR are perceived as innovative) may inflate acceptance estimates. Their findings underscore the importance of capturing patient-specific needs to inform the development of telemedicine platforms and policies [22].

Our subgroup analysis suggests targeted levers for patients: prior use conferred the largest, statistically reliable benefit among those with secondary education (Cliff’s δ = 0.56; Holm-adjusted *p* = 0.029), pointing to value in short, supervised “try-before-you-decide” experiences, transparent data notices aligned with GDPR, and integrated onboarding (e.g., one-click scheduling, clear fallback to clinician-only care). Pricing clarity and tiered options may further align willingness to pay with perceived benefit, while XR deployments should include tolerability screening and accessible alternatives to protect equity.

Moreover, our finding of moderate-to-high acceptance (Acceptance Index 3.27 ± 0.59) with no detectable between-age differences aligns with work showing broadly favorable but not uniform patient receptivity to remote and AI-inflected care. In a German primary-care survey grounded in Unified Theory of Acceptance and Use of Technology (UTAUT), overall acceptance of video consultations was also “moderate,” with performance and effort expectancy, rather than age, driving the intention to use [23]. A contemporary systematic review of video/phone telemental health also reported generally positive patient attitudes, tempered by variability across settings and outcomes, again suggesting that contextual factors (ease of use, clinical appropriateness) matter at least as much as demographics [24]. Broader public polling about AI in U.S. healthcare similarly finds cautious optimism, with interest in convenience and perceived effectiveness balanced against misgivings, consistent with our mid-to-upper scale means on trust and perceived improvement [25].

The positive association we observed between prior exposure and acceptance mirrors diffusion patterns in which experience reduces uncertainty and increases willingness to adopt. Experimental work indicates that how AI is framed also shapes support communicating accuracy and bias, especially with explicit mention of clinician oversight, increases approval for medical AI across tasks [26]. A recent scoping review of patient perspectives on AI converges on the same levers: perceived usefulness, human-in-the-loop assurance, and transparent communication are recurrent facilitators of acceptance [27]. Although privacy concerns are salient attitudinally, their link to behavior is often weak. In a large telehealth study, privacy worry had little explanatory power for continued-use intention once usability and value were accounted for, a pattern congruent with our non-association between privacy concern and acceptance or choice [28].

Willingness to pay emerged as the strongest correlate of acceptance in our multivariable models (β ≈ +0.43 per one-level increase; proportional-odds OR ≈ 6.8), highlighting the centrality of perceived value. This finding aligns with a systematic review that demonstrates a wide variation in stated willingness to pay for telemedicine, depending on the condition and context, yet it correlates with perceived convenience and benefits, particularly when telemedicine substitutes for costly travel or wait time [29]. Disease-specific studies echo this sensitivity to out-of-pocket framing: in a telemedicine lifestyle program, most participants were willing to pay modest copays (<US$30), with acceptance rising as travel burdens increased [30]. From a system perspective, economic evaluations of digital health interventions highlight that uptake hinges not only on clinical performance but also on clear pricing and cost offsets (e.g., fewer visits, reduced transportation), which help patients and payers reconcile value with fees [31].

Accessibility also showed an independent association with choosing an AI-assisted visit (OR ≈ 1.83), which is consistent with evidence that lowering frictions, devices, connectivity, and onboarding shifts preferences toward virtual care. Therefore, reducing practical barriers—simpler log-in and scheduling, loaner devices where needed, reliable broadband or cellular alternatives, and brief guided first-use tutorials—can shift preferences toward virtual care. In the VA program that mailed video-enabled tablets to veterans with access barriers, two-thirds of recipients preferred or were indifferent to tablet-based visits relative to in-person care, citing fewer logistics and smoother access as key reasons [32]. A subsequent evaluation of the VA’s Digital Divide Consult found that targeted device distribution and support significantly boosted telehealth adoption among patients lacking access to technology or broadband, reinforcing the idea that “facilitating conditions” are leverage points for acceptance in real-world populations [33].

Lastly, our bundled AI/XR framing is consistent with emerging XR evidence suggesting generally good acceptability when content is purposeful and tolerability is managed. Moreover, acceptability is high when tolerability is actively managed—for example, by limiting motion-rich content, offering seated modes, shorter initial sessions, and easy opt-out. In mixed chronic pain cohorts, VR interventions demonstrate high user satisfaction and acceptance, alongside clinically meaningful symptom improvements [34]. Educational and preparatory use cases, such as VR walk-throughs before radiotherapy, demonstrate feasibility and reductions in anxiety, with encouraging patient-reported value [35]. VR walk-throughs’ refers to short, immersive previews that simulate a care pathway (e.g., radiotherapy setup) to set expectations and reduce anxiety.

Oncology feasibility studies similarly report high acceptability of immersive programs during chemotherapy [36]. In oncology, immersive programs typically deliver brief relaxation, mindfulness, or distraction modules via a headset during infusion sessions. On the technical edge, reviews of augmented reality for real-time telemedicine note usability gains but emphasize workflow fit and device ergonomics as prerequisites for sustained use [37], while implementation scoping reviews across XR catalog common barriers, cost, staff training, motion sensitivity, and recommend gradual onboarding and opt-in pathways, strategies that resonate with our secondary-education subgroup where prior exposure had the largest acceptance gain [38]. Gradual onboarding’ means starting with optional, short trial sessions and step-ups only if tolerated; ‘opt-in pathways’ means patients choose XR components explicitly, with standard telemedicine as a default alternative. Nevertheless, our instrument did not capture clinical condition or acuity; such contextual factors may materially shape preferences and should be incorporated in future designs.

Future studies should (i) validate the Acceptance Index with factor-analytic methods and test–retest reliability; (ii) experimentally separate AI versus XR attributes (e.g., discrete-choice designs varying accuracy, explainability, comfort, and cost); (iii) include objective usage metrics alongside stated preferences; (iv) evaluate equity-focused facilitation (loaner devices, guided onboarding) on acceptance; and (v) assess clinical-condition effects on preferences to tailor telemedicine pathways.

### 4.2. Study Limitations

This cross-sectional, self-reported, web-based survey is susceptible to selection bias, common-method bias, and social desirability effects; the sample is skewed toward mid-life, highly educated respondents, which limits generalizability. Small cell sizes at older ages reduce power to detect age effects. The Acceptance Index, although internally consistent, aggregates distinct constructs and lacks external validation; we did not measure digital literacy, broadband quality, health status, or clinical use contexts that could confound the associations. We did not assess age-related differentials in prior AI/XR exposure, which may confound age–acceptance patterns; future work should stratify exposure by age. Because the instrument bundled AI and XR, we could not test whether XR-specific awareness further elevates willingness to pay beyond AI alone. Discrete-choice experiments that vary AI versus XR features could quantify any incremental WTP. We also could not isolate any incremental effect of having access to XR features on the choice of an AI-assisted visit; modality-specific effects remain to be tested. Finally, our instrument assessed stated acceptance and choice rather than observed behavior, and it bundled AI and XR in a single frame, potentially masking modality-specific attitudes.

## 5. Conclusions

Patients in this Romanian cohort expressed moderate to high acceptance of AI/XR-enabled telemedicine, with acceptance most strongly linked to perceived value (willingness to pay; OR = 6.8) and ease of access (OR = 1.8); however, privacy concerns did not significantly diminish acceptance. Prior exposure was associated with higher acceptance overall and showed the largest confirmed benefit among those with secondary education, suggesting that optional, low-stakes exposure pathways, such as a 5–10 min supervised demo, an optional first visit with clinician override, and clear ‘return to standard visit’ options at any time, could accelerate informed uptake. Implementation should therefore prioritize clear communication of benefits and limits, streamlined onboarding with accessible fallbacks (including phone/video without AI features, non-immersive education modules, and asynchronous messaging when bandwidth or tolerability is limited), pricing transparency, and XR design that minimizes cybersickness. Within the EU AI Act/GDPR context, trustworthy data handling and post-market monitoring are prerequisites for sustained patient confidence. Future work should validate the Acceptance Index, incorporate objective usage metrics, and test causal levers, such as randomized “opt-in AI assist,” usability improvements, and pricing frames, across different segments to ensure equitable and patient-centered deployment.

## Figures and Tables

**Table 1:** Sample characteristics.

Section	Category	n	%
Age Group	21–30 years	39	19.7
	31–40 years	63	31.8
	41–50 years	61	30.8
	51–60 years	24	12.1
	61–70 years	2	1
	71–80 years	9	4.5
Education Level	Primary education	5	2.5
	Secondary education	42	21.2
	Undergraduate degree	104	52.5
	Postgraduate (Master/PhD)	47	23.7
Used AI/XR Services	Yes	54	27.3
	No	118	59.6
	Don’t know	26	13.1
Knowledge Level	Not informed	29	14.6
	Slightly informed	76	38.4
	Moderately informed	72	36.4
	Very informed	21	10.6

**Table 2:** Acceptance metrics by age group.

Age Group	Trust in AI	Perceived Improvement	Choose AI Visit	Acceptance Index
21–30	3.00 (0.73)	3.46 (0.72)	3.38 (0.67)	3.28 (0.60)
31–40	3.02 (0.63)	3.49 (0.72)	3.17 (0.81)	3.23 (0.62)
41–50	3.20 (0.77)	3.46 (0.67)	3.18 (0.74)	3.28 (0.62)
51–60	3.17 (0.64)	3.38 (0.77)	3.25 (0.61)	3.26 (0.54)
61–70	3.00 (0.00)	3.50 (0.71)	3.00 (1.41)	3.17 (0.71)
71–80	3.33 (0.50)	3.67 (0.71)	3.44 (0.53)	3.48 (0.34)

Kruskal–Wallis *p*-values: Trust 0.4565; Perceived improvement 0.8746; Choose AI visit 0.7111; Acceptance Index 0.9216.

**Table 3:** Acceptance by prior AI/XR use (Mann–Whitney U).

Group	n	Mean (SD)
Used AI/XR (Yes)	54	3.47 (0.47)
Used AI/XR (No)	118	3.19 (0.59)

U = 4157.5; *p* = 0.0011 (two-sided).

**Table 4:** Spearman correlations among key variables.

	Knowledge	Privacy Concern	Accessibility	Acceptance
Knowledge	1	0.259 (*p* = 0.0063)	0.322 (*p* = 0.0006)	0.189 (*p* = 0.0478)
Privacy concern	0.259 (*p* = 0.0063)	1	0.141 (*p* = 0.1421)	0.072 (*p* = 0.4547)
Accessibility	0.322 (*p* = 0.0006)	0.141 (*p* = 0.1421)	1	0.229 (*p* = 0.0162)
Acceptance	0.189 (*p* = 0.0478)	0.072 (*p* = 0.4547)	0.229 (*p* = 0.0162)	1

**Table 5:** Privacy concern × choosing an AI-assisted visit.

High Privacy Concern	No/Low Choice (1–2)	Yes/Maybe (3–4)
No	14 (16.5%)	71 (83.5%)
Yes	3 (12.0%)	22 (88.0%)

χ^2^ = 0.052; df = 1; *p* = 0.819.

**Table 6:** OLS regression predicting the Acceptance Index.

Predictor	Beta	SE	*p*-Value
Intercept	2.427	0.636	<0.001
Knowledge (1–4)	−0.048	0.074	0.513
Privacy concern (1–3)	−0.131	0.133	0.325
Accessibility (1–4)	0.078	0.077	0.308
Willing to pay more (1–4)	0.432	0.068	<0.001
Age 31–40 (comparison 21–30)	−0.230	0.144	0.111
Age 41–50	−0.030	0.132	0.821
Age 51–60	0.04	0.191	0.833
Age 61–70	−0.065	0.755	0.931
Age 71–80	0.115	0.238	0.629
Secondary education (comparison Primary)	−0.221	0.441	0.617
Postgraduate	−0.047	0.433	0.913
Undergraduate	−0.069	0.433	0.873
Prior use = No (comparison Yes)	−0.104	0.124	0.401
Prior use = Don’t know	−0.005	0.231	0.983

HC3 robust SE; n = 110; R^2^ = 0.461.

**Table 7:** Subgroup analysis: Prior AI/XR use within education strata.

Education Level	n (Yes)	Mean ± SD (Yes)	n (No)	Mean ± SD (No)	U-Stat	*p* (Raw)	Cliff’s δ	*p* (Holm)
Primary education	0	NA	4	3.17 (0.33)	—	—	—	—
Secondary education	10	3.43 (0.39)	24	2.78 (0.75)	187.5	0.0097	0.563	0.0291
Undergraduate degree	30	3.43 (0.55)	63	3.26 (0.54)	1161	0.0697	0.229	0.1394
Postgraduate (Master/PhD)	14	3.57 (0.33)	27	3.38 (0.41)	237	0.174	0.254	0.174

Prior use associates with higher acceptance across education tiers and remains significant after Holm correction in the secondary education stratum (moderate effect, Cliff’s δ ≈ 0.56).

**Table 8:** Ordinal logistic regression (proportional-odds) for choosing an AI-assisted visit (1–4).

Predictor	OR	95% CI	*p*-Value
Knowledge (1–4)	1.214	0.68–2.17	0.514
Privacy concern (1–3)	0.492	0.16–1.56	0.228
Accessibility (1–4)	1.826	1.03–3.24	0.04
Willing to pay more (1–4)	6.807	3.39–13.66	<0.001
Prior use (Yes = 1)	0.63	0.21–1.90	0.412
Age (ordinal 1–6)	1.085	0.76–1.56	0.659
Education (ordinal 1–4)	0.815	0.44–1.50	0.512

## Data Availability

The data presented in this study are available on request from the corresponding author. The data are not publicly available due to privacy and ethical restrictions.
